# PD-1 Inhibitor Therapy in a Patient with Preexisting P-ANCA Vasculitis: A Case Report and Review of the Literature

**DOI:** 10.1155/2020/3428945

**Published:** 2020-08-31

**Authors:** Amanda Ramos, Marcela del Carmen, Oladapo Yeku

**Affiliations:** ^1^Division of Gynecologic Oncology, Department of Obstetrics and Gynecology, Hartford Healthcare, Hartford, CT 06102, USA; ^2^Division of Gynecologic Oncology, Vincent Obstetrics and Gynecology, Harvard Medical School, Boston, MA 02114, USA; ^3^Gynecologic Cancers Program, Massachusetts General Hospital, Harvard Medical School, Boston, MA 02114, USA

## Abstract

**Background:**

Recurrent endometrial cancer after definitive therapy is a lethal disease. Recently, immune checkpoint inhibitors (ICI) have improved the management of mismatch repair-deficient (MSI-H) endometrial cancer. Autoimmune side effects are known to occur with ICI. As a result, patients with preexisting autoimmune diseases are excluded from studies involving these drugs. This has led to challenges in clinical practice regarding the use of ICI in otherwise eligible patients with underlying autoimmune disease. *Case Presentation*. We present the case of an 81-year-old woman with an underlying autoimmune vasculitis and recurrent, metastatic endometrial adenocarcinoma with microsatellite instability, who was treated with an immune checkpoint inhibitor. This patient received pembrolizumab, an immune checkpoint inhibitor that targets the programmed cell death-1 immune checkpoint. Ultimately, she was treated for 4 months with pembrolizumab and benefited from stable disease during this period. She remained asymptomatic from her underlying autoimmune P-ANCA vasculitis. A review of the scientific literature reveals several cases of the successful use of immune checkpoint inhibitors in patients with autoimmune diseases, including systemic lupus erythematosus, rheumatoid arthritis, and inflammatory bowel disease.

**Conclusion:**

This is one of the first reports of a patient with an underlying autoimmune vasculitis successfully treated with an immune checkpoint inhibitor without exacerbating her underlying autoimmune condition. Carefully selected patients with underlying autoimmune vasculitis can be safely treated with ICI.

## 1. Introduction

Recurrent endometrial cancer poses a treatment challenge for both patients and clinicians. Of the approximately 60,000 women who are diagnosed annually with endometrial cancer, about 15% will recur following first-line therapy, the vaginal apex being the most common location for recurrent disease [1, 2].

Immune checkpoint inhibitors (ICI) have revolutionized the treatment of numerous solid tumor malignancies, such as nonsmall cell lung cancer, melanoma, and renal cell carcinoma [3, 4]. Immune checkpoints such as programmed death receptor-1 (PD-1) are transmembrane proteins that are expressed on activated or exhausted cytotoxic T-cells and help prevent against autoimmunity under normal circumstances [5]. When tumor-infiltrating lymphocytes (TILs) recognize an antigen and become activated, PD-1 binds to its natural ligand, programmed death receptor-ligand 1 (PD-L1) expressed on tumor cells, leading to the suppression of cytotoxic T-cell activity. This pathway evolved as a counter-regulatory mechanism to limit inflammation and damage to healthy tissue. Unfortunately, this pathway is also co-opted by tumor cells to evade the immune system [5]. Therapeutic PD-1 blockade inhibits tumor-mediated T-cell suppression but also increases the risk of autoimmunity.

Anti-PD-1 therapy has been evaluated in several solid tumor malignancies. Le et al. [6] reported a 53% objective response rate in patients with mismatch repair-deficient (MSI-H/MMR) endometrial cancer [6]. This led to the FDA approval of pembrolizumab (Keytruda®), an anti-PD-1 ICI, in MSI-H/MMR solid tumors including endometrial cancer. Follow-up studies of pembrolizumab in mismatch repair intact or microsatellite stable (MSS) endometrial cancer reported a 13% objective response rate [7]. For comparison, single-agent cytotoxic chemotherapy has response rates ranging from 4% to 27% in the recurrent setting [8].

Patients with preexisting autoimmune disease have historically been excluded from clinical trials involving ICI. However, as real-world adoption of ICI increases, physicians face the decision of whether or not to use ICI in otherwise suitable patients with underlying autoimmune disease. Unfortunately, there is limited information regarding the safety and efficacy of ICI that can be used to guide treatment decisions in this patient population.

In this report, we present the case of a patient with underlying p-ANCA vasculitis and recurrent mismatch repair-deficient (MSI-H/MMR) endometrial adenocarcinoma treated with pembrolizumab without exacerbation of her underlying autoimmune disease.

## 2. Case Presentation

The patient is an 83-year-old woman with a history of major depressive disorder, psoriasis, hypertension, and coronary artery disease who presented to our institution in July of 2017 with postmenopausal bleeding. Pelvic ultrasonography performed on September 19, 2017, revealed a diffusely thickened endometrium. An endometrial biopsy revealed grade 1 of 3 endometrioid adenocarcinoma. As a result of these findings, the patient underwent a total laparoscopic hysterectomy, bilateral salpingo-oophorectomy, and lysis of adhesions on August 22, 2017. Intraoperative findings were notable for tumor protruding from the cervical os. On laparoscopic examination, there was no evidence of extrauterine disease. Given the patient's advanced age and extensive adhesive disease, lymph node dissection was omitted.

The final pathology from this procedure was reviewed, revealing a FIGO grade 2 of 3 endometrioid adenocarcinoma of the endometrium measuring 8.4 cm with 18.5 mm of invasion into a 19 mm thick myometrium. Other pathologic findings included lymphovascular space invasion, cervical stromal invasion, and parametrial involvement. Notably, necrotizing arteritis was identified in her adnexa bilaterally in a manner that was substantially more than expected in routine oophorectomy specimens. Immunohistochemistry revealed a loss of MLH1 and PMS2, with further analysis showing MLH1 promoter methylation, consistent with MSI-H/MMR.

One month following her surgery, she was evaluated by her rheumatologist for further workup of the necrotizing arteritis found in her adnexa. Serologic analysis revealed that she was positive for perinuclear antineutrophil cytoplasmic antibody (MPO-ANCA) consistent with p-ANCA vasculitis. Treatment was deferred as she had no other evidence of active vasculitis.

She received adjuvant external beam radiation therapy to the pelvis for a total of 4500 cGy, followed by high dose rate brachytherapy to a total of 700 cGy over three fractions. At the completion of her therapy in December of 2017, she was admitted to the hospital with C. difficile colitis and acute kidney injury with a creatinine of 3.17 mg/dL (reference range; 0.60-1.50 mg/dL). Her creatinine continued to rise despite fluid resuscitation for what was initially considered prerenal azotemia. A repeat CT scan was performed, and this revealed evidence of interstitial lung disease. In the context of her positive p-ANCA titers and history of adnexal vasculitis, the decision was made to treat her for systemic p-ANCA vasculitis. She received solumedrol, rituximab, cyclophosphamide, and plasmapheresis with rapid improvement of her creatinine to baseline prior to discharge. After induction treatment, her steroids were tapered off, and she continued to receive maintenance rituximab.

**Figure 1:**
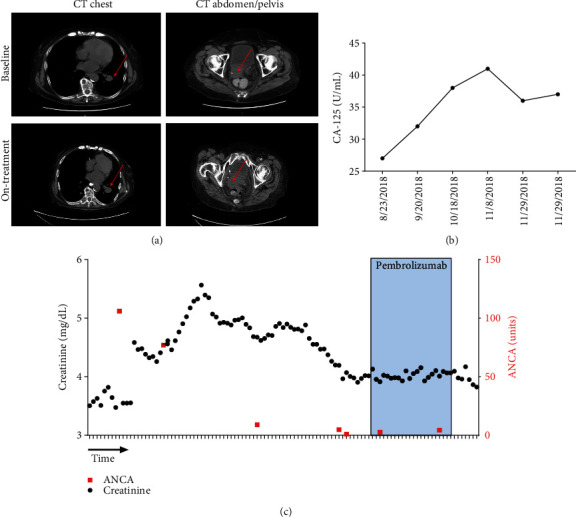
Pembrolizumab monotherapy in a patient with underlying p-ANCA vasculitis describes the stability of this patient's malignancy as well as the stability of her underlying autoimmune vasculitis while on pembrolizumab therapy. (a) displays computerized tomography (CT) images at baseline and on-treatment after 3 cycles of pembrolizumab. The size of the vaginal apex mass and lung nodules remained relatively unchanged. Repeat CT scan of the chest, abdomen, and pelvis revealed a pulmonary nodule (1.0 cm), an enlarged perihilar lymph node (1.4 cm), and a right-sided vaginal apex mass (2.8 cm) causing right-sided hydronephrosis (a). (b) CA-125 tumor marker levels are shown. (c) Serum creatinine level over time (black circles, left axis) and ANCA levels (red squares, right axis) are shown. Time on pembrolizumab is highlighted.

She was lost to follow-up for several months and returned in August of 2018 for evaluation of vaginal bleeding. Physical examination revealed the presence of a vaginal apex mass that was biopsy-proven to be recurrent endometrial adenocarcinoma. Given her MSI-H/MMR and her inability to tolerate platinum-based cytotoxic chemotherapy due to her poor performance status, the decision was made to start pembrolizumab 200 mg i.v. every three weeks. She underwent three cycles followed by restaging CT scans which showed stable disease (Figure 1(a)).  Figure 1(c) displays the patient's creatinine levels (black circle, left *y*-axis) and ANCA (red square, right *y*-axis) plotted over time. Time on pembrolizumab is highlighted in blue. During this treatment period, her renal function was followed closely (Figure 1(c)), and urinalysis was obtained frequently to evaluate for hematuria. She did not develop any signs or symptoms suggestive of a vasculitis flare.  Figure 1(b) displays the patient's CA-125 values over the same time period. Despite her stable CA-125 (Figure 1(b)) and radiographically stable disease (Figure 1(a)), the patient gradually became more socially withdrawn, depressed, and decreased her oral intake despite maximal support from her family and treatment by her psychiatrist. The decision was made to stop cancer-directed care and provide comfort measures only. The patient died on December 18, 2018, four months after starting pembrolizumab.

## 3. Discussion

In this report, we present the case of a patient with underlying p-ANCA vasculitis and recurrent MSI-H endometrial cancer successfully treated with anti-PD-1 immune checkpoint blockade. While this patient did have an underlying autoimmune disorder, the administration of pembrolizumab did not exacerbate her underlying autoimmune condition. Furthermore, she did not experience any immune-related adverse events, and she had stable disease as her best response to treatment. To our knowledge, this is the first report of a patient with underlying p-ANCA vasculitis, and recent treatment with plasmapheresis, prednisone, cyclophosphamide, and rituximab, who was safely treated with pembrolizumab.

Common immune-related adverse events associated with ICI include rash, hypophysitis, hepatitis, hypothyroidism, type-1 diabetes, arthritis, colitis, and pneumonitis [9]. Of these, pneumonitis is the most severe adverse effect seen with PD-1 inhibition [10]. Studies investigating the pathogenesis of immune-mediated toxicities have revealed the presence of T-cell and macrophage infiltration into affected tissues, as well as antibody-dependent complement deposition [11, 12]. There have been at least two documented cases of de novo vasculitis associated with ICI. Cases of giant cell arteritis and polymyalgia rheumatica were documented in 2 case reports after CTLA-4 inhibitor therapy with ipilimumab (Yervoy®) for advanced melanoma [13]. Cases of retinal and uterine vasculitis following ipilimumab and pembrolizumab have also been reported [12–15]. Often, these toxicities result in the discontinuation of therapy.

The antineutrophil cytoplasmic antibody-associated vasculidities are comprised of granulomatosis with polyangiitis, microscopic polyangiitis, and eosinophilic granulomatosis with polyangiitis. These vasculidities are characterized by the presence of autoantibodies directed at neutrophil cytoplasmic constituents such as proteinase-3 and myeloperoxidase [16, 17]. The patient presented in this case study was diagnosed with a myeloperoxidase antineutrophilic cytoplasmic antibody-associated vasculitis (MPO-ANCA), which is a small-vessel vasculitis [18]. PD-1 has been shown to be expressed on B-cells [19]; however, its function in shaping the immune tumor microenvironment has only recently been elucidated [19]. It is theoretically possible that PD-1 inhibition on B-cells could exacerbate an underlying antibody-mediated vasculitis. Patients with preexisting autoimmune conditions such as ANCA-associated vasculitis are excluded from most ICI clinical trials given the concern for increased activation of the immune system and increased risk of inducing or exacerbating autoimmune diseases [20].

Several small retrospective studies have reported exacerbation of underlying autoimmunity by ICI therapy. Johnson et al. [21] reported on 52 patients across 13 medical centers with preexisting autoimmune diseases including rheumatoid arthritis (RA), systemic lupus erythematosus (SLE), scleroderma, and inflammatory bowel disease, who underwent treatment with ipilimumab. Twenty-seven percent (13 patients) experienced an exacerbation of their underlying autoimmune disease. These flares were effectively treated with corticosteroids. Of note, 50% of patients in this study did not experience any immune-related adverse events [21]. In another review of 30 patients across nine medical centers with preexisting autoimmune disease including multiple sclerosis, sarcoidosis, RA, and SLE, treated with ICI, approximately 50% experienced a flare in their underlying disease symptoms [15]. Kehl et al. [20] identified 462 patients with preexisting autoimmune conditions treated with ipilimumab, nivolumab (Opdivo®), pembrolizumab, or atezolizumab (Tecentriq®). In this series, there was no association between immune checkpoint therapy and all-cause hospitalization. However, the authors concluded that patients with preexisting autoimmune disease were more likely to develop immune-related adverse events and, as a result, receive corticosteroids. Yoneshima et al. [22] examined the effects of PD-1 inhibitors on patients with preexisting ANA positive titers. There was no increase in the incidence of adverse events in this patient population compared to patients with negative ANA [22]. However, patients with rising ANA titers on therapy were found to have an increased incidence of grade 3-5 irAEs. This study supports the notion that patients with preexisting autoimmune diseases may receive ICI with close monitoring [22].

There are limited data in patients with preexisting autoimmune vasculitis. A case report by Maul et al. described a patient with preexisting Churg-Strauss disease treated with ICIs [23]. This patient was successfully treated with ipilimumab, followed by the initiation of pembrolizumab monotherapy after the development of colitis secondary to ipilimumab. Since the patient's initial diagnosis of Churg-Strauss, he had been treated with maintenance prednisolone, and this continued while on treatment with ipilimumab [23]. It is possible that, in this case, maintenance prednisolone may have prevented a flare of his underlying condition.

This case report adds to the literature regarding the safety of anti-PD-1 immune checkpoint inhibition in patients with underlying autoimmune disease. This patient with preexisting p-ANCA vasculitis was successfully treated with pembrolizumab (Keytruda®), an anti-PD-1 ICI, without exacerbation of her underlying vasculitis. Additionally, the patient experienced stable disease, an acceptable treatment outcome in elderly patients with incurable cancer, and comorbid medical conditions. Although caution should be taken when treating patients with stable underlying autoimmune disorders with ICIs, flares are not as common as fared, and patients who do develop flares can be readily treated with corticosteroids provided the symptoms are recognized early enough.

## Abbreviations

CT: Computerized tomography
ICI: Immune checkpoint inhibitors
TILs: Tumor-infiltrating lymphocytes
PD-1: Programmed death receptor-1
PD-L1: Programmed death receptor-ligand 1
MSI-H/MMR: Mismatch repair-deficient
MSS: Microsatellite stable.
